# Evaluation of the usability of the spinal cord stimulator recharging procedure in the treatment of neuropathic pain. A quantitative and qualitative study

**DOI:** 10.3389/fpain.2026.1727882

**Published:** 2026-05-11

**Authors:** Delphine Durand, Pierre-Henri Garnier, Sylvain Durand, Julien Nizard, Vincent Wyart, Céline Brouillet, Jean-Pascal Lefaucheur, Jean-Paul Nguyen

**Affiliations:** 1 Centre d'Evaluation et de Traitement de la Douleur (CETD), Clinique Bretéché, Elsan Group, Nantes, France; 2 Service de Psychiatrie de l’enfant et de l’adolescent, CHU de Nantes, France; 3 Centre d'Evaluation et de Traitement de la Douleur (CETD), Soins Palliatifs et de Support, CHU de Nantes, France; 4 UMR 1229 (Médecine Regénératrice et Squelette), INSERM, Université de Nantes, Nantes, France; 5 Santé Atlantique, Elsan Group, Nantes, France; 6 UR 4391, Team ENT (Nerve Excitability and Therapeutics), Université Paris-Est Créteil, Créteil, France; 7 Clinical Neurophysiology Unit, Henri Mondor University Hospital, Assistance Publique—Hôpitaux de Paris (AP-HP), Créteil, France

**Keywords:** neuropathic pain, qualitative analysis, rechargeable stimulators, spinal cord stimulation, usability scale

## Abstract

**Background:**

Rechargeable stimulators have improved the management of patients treated with spinal cord stimulation by avoiding numerous interventions to replace stimulator devices whose batteries are reaching the end of their life. However, the recharging procedure can be restrictive for some patients and affect their satisfaction.

**Objective:**

The aim of this work is to evaluate the usability of the recharging procedure through a quantitative and qualitative approach to explore the experience of unsatisfied patients.

**Methods:**

The evaluation began with a retrospective analysis of 50 patients with neuropathic pain who have had a rechargeable spinal cord stimulator for more than one year. Patients completed the French version of the System Usability Scale (F-SUS) which is a scientifically validated usability questionnaire. Ten patients who were not satisfied with the recharging procedure (F-SUS score <70) has been interviewed and their speech has been analysed thematically.

**Results:**

These were 26 women and 24 men, mean age 51.6 years, operated on in 2019 and 2020 for spinal cord stimulation using a rechargeable stimulator for the treatment of neuropathic pain refractory to medical therapy. Forty patients (80% of cases) were satisfied (F-SUS score ≥70/100) and 10 patients not satisfied (F-SUS score <70) with the recharging procedure. The numerical pain scale improved by an average of 52% over the long term (mean follow-up 3.6 years). Rechargement time averaged 59.6 min (±35) and the interval between rechargement sessions 4.2 days (±7.1). These data were not significantly different between satisfied and not satisfied patient groups. The qualitative study revealed that the duration of the rechargement session was the main cause of dissatisfaction among unsatisfied patients. This is linked to the fact that these patients must remain virtually motionless throughout the session, otherwise recharging is interrupted by loss of contact between the recharging box and the stimulator. This study also highlights the fact that patients who have improved well with spinal cord stimulation are better able to tolerate the inconvenience of recharging, which is not the case for patients whose pain has improved little or not at all. Overall, patients would like to be better prepared for the recharging procedure.

**Conclusion:**

The mixed quantitative and qualitative analysis indicates that most patients are satisfied with the recharging procedure. The problem of contact between the recharging device and the stimulator device needs to be improved, as it is the main cause of dissatisfaction among patients who are not satisfied with the recharging procedure.

**Ethics and dissemination:**

This study was approved by the French ethics committee (CPP Est I ID RCB: 2022-A00683-40) on June 3, 2022, and registered on ClinicalTrials.gov (NCT05373654) on May 9, 2022.

## Background

Neuropathic pain is linked to the presence of one or more lesions of the central or peripheral nervous system (1). In France, their prevalence in the general population was evaluated at 31.7% in the 2008 STOPNEP survey (“Study of Prevalence of Neuropathic Pain”) (2). In general, neuropathic pain becomes resistant to drug treatments and neurostimulation techniques are increasingly used (1). Among them, spinal cord stimulation (3) which is now recommended in France by the Haute Autorité de Santé (HAS) for the relief of patients suffering from refractory chronic neuropathic pain (4) located in the trunk, upper or lower limbs. This technique has been shown to be very effective, improving more than 75% of patients, in the treatment of neuropathic pain related to nerve root injury (sequelae of cervical or lumbar disc herniation surgery) (5), peripheral nerves lesions (surgical or traumatic injuries) or from a Complex Regional Pain Syndrome (6). It has also been shown that spinal cord stimulation can improve arterial circulation and lower limb pain in patients with atheromatous arteritis (7).

One of the disadvantages of spinal cord stimulation is the limited life of the batteries contained within standard stimulators. The average battery life has been estimated at 3.7 years (8). This means that the stimulator must be replaced very frequently. To overcome this problem, rechargeable stimulators have been developed and have been in routine use since 2010. Some rechargeable stimulators have a fixed life span of 9 years, while others theoretically have an unlimited life span. For the moment, their cost is relatively high. A rechargeable stimulator currently costs approximately 3 times more than a standard stimulator.

Now, with the benefit of more than 10 years' experience of using rechargeable spinal cord stimulators, the question arises as to whether to use a standard or rechargeable stimulator. There is an undeniable economic advantage to using a rechargeable stimulator. Over 10 years, the cost of a rechargeable stimulator implantation in the context of spinal cord stimulation is approximately 5–6 times less expensive than the cost of multiple implantations of a standard stimulator (8–10). However, the ease of use of the systems in everyday life should also be considered in this choice. With a standard stimulator, some patients do not have to take care of the stimulation which they leave on permanently. So, there is no constraint. Some patients will modulate the intensity of the stimulation with a remote control by reducing or stopping it when they are not in pain, which extends the life of the batteries. In the past it was also often necessary to lower the stimulation intensity when changing to a lying position, but most stimulators now incorporate a system for automatically adjusting the stimulation intensity when changing position. Similarly, cervical spinal cord stimulation can trigger paraesthesia's when the neck is moved.

In our team, rechargeable stimulators are implanted as a first-line treatment if the pain has only been improved by using high stimulation intensities (>5 volts or >5 mA at an impedance of about 1000 ohms) during the test period (at least one week) suggesting that stimulation methods that consume a great deal of power (high density and high frequency) (3) could be used. As a second line of treatment, a rechargeable stimulator is offered as a replacement for a standard stimulator if the life of the latter has been less than 3 years.

The operating constraints are greater when using a rechargeable stimulator because the stimulator must be recharged regularly. To recharge, the patient places an antenna on the skin above the stimulator. The antenna is connected to the charging device, which has been previously charged from the mains. For charging to be easy, the tissue between the antenna and the stimulator should not be too thick and the position of the antenna in relation to the stimulator should be as fixed as possible throughout the charging process, which is facilitated by a belt that holds the antenna in position. When all the conditions are met, the battery recharge (the patient is generally advised to fully recharge as soon as only 50% of the battery capacity remains) can take up to 2 h. When the stimulation intensities remain in “normal” zones (<5 volts), the patient recharges on average once a week, which is compatible with almost normal daily activities (11). The constraints become more important, even unbearable in the medium and long term, if recharging takes longer and has to be repeated at short intervals, which happens in about ¼ of patients (11, 12). Moreover, with the usual rechargeable stimulator models it is necessary to keep the stimulator constantly charged as a full discharge usually results in the impossibility of recharging, requiring an intervention to replace the stimulator.

Questionnaires dedicated to evaluating the usability of rechargeable stimulators have been proposed in areas concerning spinal cord stimulation (11–13), the treatment of headache by occipital nerve stimulation (14) or the treatment of abnormal movements by deep brain stimulation (15). The results show that about 25% of patients are not satisfied with a rechargeable stimulator system (11–13). However, none of these questionnaires have been scientifically validated, a fortiori in a French version. On the other hand, the System Usability Scale (SUS) has been validated for a long time (16, 17), and has recently been validated in its French version (F-SUS) (18).

In this article, we report a quantitative study, using the F-SUS questionnaire, focusing on the practical problem of rechargeable stimulators used for more than one year for spinal cord stimulation treatment at the Bretéché clinic for 2 years (2019–2020) by the same operator (SD). In addition, we also report a qualitative assessment of the recharging procedure, which has never been done before.

## Quantitative study

### Patients and methods

In 2019 and 2020, 160 patients had a rechargeable spinal cord stimulator implanted in our centre. Patients were contacted by telephone and if they agree, questionnaires and a consent form to be signed has been sent to them by post. The questionnaires consist of the F-SUS and a pain rating scale. Patients will also be asked how often they recharge on average and the average duration of the recharge. We felt that 50 patients were sufficient to obtain interpretable results, mainly because the patient cohorts published on this subject comprised only between 25 and 41 patients (see discussion). The F-SUS questionnaire uses the Likert scale with 5 possible answers ranging from “Strongly disagree” to “Strongly agree” (19). It is a short questionnaire (see Appendix) with 10 questions (17). In its original form, half of the questions express strong agreement and half express disagreement. Thus, all even-numbered items (2, 4, 6, 8, 10) express a very negative opinion (disagreement). Conversely, the odd-numbered items allow a very positive opinion to be expressed (strong agreement). This arrangement has the advantage of avoiding positivity bias, as some individuals are more likely to respond positively than negatively. In the same way, systematic opposition bias is avoided. This alternation of negative and positive tones should also oblige candidates to read the wording carefully and to try to think before giving their answers (20). Item 1 (“I would like to use this procedure frequently”) of the F-SUS questionnaire is not very suitable for the evaluation of the recharging procedure as this item rather assesses the hedonic side and/or attractiveness of the procedure (21). Indeed, the frequency of use of the procedure is here dictated by the battery charge level and in principle not by the patient's pleasure or the attractiveness of the procedure. All studies have validated the F-SUS questionnaire on a unidimensional scale (18). It therefore seemed essential to us to keep this item in the questionnaire. It has been shown that changing the wording of an item, or even reversing the tone of the response (positive or negative tone of the question) does not influence the final score, provided that the meaning of the item is retained (16, 22). The general meaning of the wording of item 1 is the pleasure of use (hedonic quality) and/or the pleasantness (overall attractiveness) of the procedure. The following pairs of words seem to be the most appropriate to express the meaning of the wording of item 1: (i) Pleasant/unpleasant, (ii) agreeable/disagreeable (23). A negative tone seemed to us to be the most appropriate, which is why we retained the following wording: “I find this procedure unpleasant”. If the patient remains undecided or does not fully understand the meaning of the question, he or she is advised to use a neutral modality by answering 3 to the question because in the SUS questionnaire there is no “don’t know” box (24, 25). The scores of the inverted items of this modified version, expressing disagreement (1, 2, 4, 6, 8, 10) are put back in the right direction thanks to the way the global score of the F-SUS is calculated (see Appendix). The maximum total score is 100, which will be used for the different analyses. Thus, satisfaction is considered good from a score of 70 and excellent from 90. Satisfaction is considered correct between 50 and 70, but improving the system is needed. Below a score of 50, the system is considered unusable (26). We will consider satisfied patients to have an F-SUS score of 70/100 or higher. This will define the groups of satisfied and dissatisfied patients.

The improvement in pain intensity secondary to spinal cord stimulation has been studied by the numerical pain rating scale (NRS), from 0 (no pain) to 100 (maximum possible pain). The preoperative NRS score was calculated as the average of the scores recorded three times during the day (morning, midday and evening) prior to the procedure. As with the preoperative NRS values, we asked patients to record their NRS values in the morning, at midday and in the evening on the day before they completed the questionnaire. Pain improvement was assessed both in the short term (3 months after surgery) and in the long term (mean follow-up of 3.6 years +/− 0.4 ranging from 2.8 to 4.3 years).

Patients were asked about their % satisfaction with the use of their rechargeable stimulator.

Recharge parameters were measured at the time of evaluation (questionnaire): recharged duration in minutes and interval between recharges in days.

The stimulation parameters used were those recorded during the last stimulator adjustment: Amplitude [milliampere (mAmp)], frequency (Hertz), pulse width (microseconds). They had not been modified up to the evaluation date. The current stimulation mode was noted: tonic, high frequency (HF) and high density (HD). There were no patients in burst mode, as they were not using rechargeable stimulators.

### Statistical analysis

The significance threshold was set at 5%. A comparison between the 2 groups (F-SUS score ≥70 and F-SUS score <70) was performed using the non-parametric Mann–Whitney test for pre and post operative (short and long-term) numeric pain score. A comparison between the 2 groups regarding different parameters of recharging and stimulation was also performed using the same statistical test [XLSTAT statistical and data analysis solution. https://www.xlstat.com/fr. Lumivero (2025). Paris, France].

## Results

**Figure 1:**
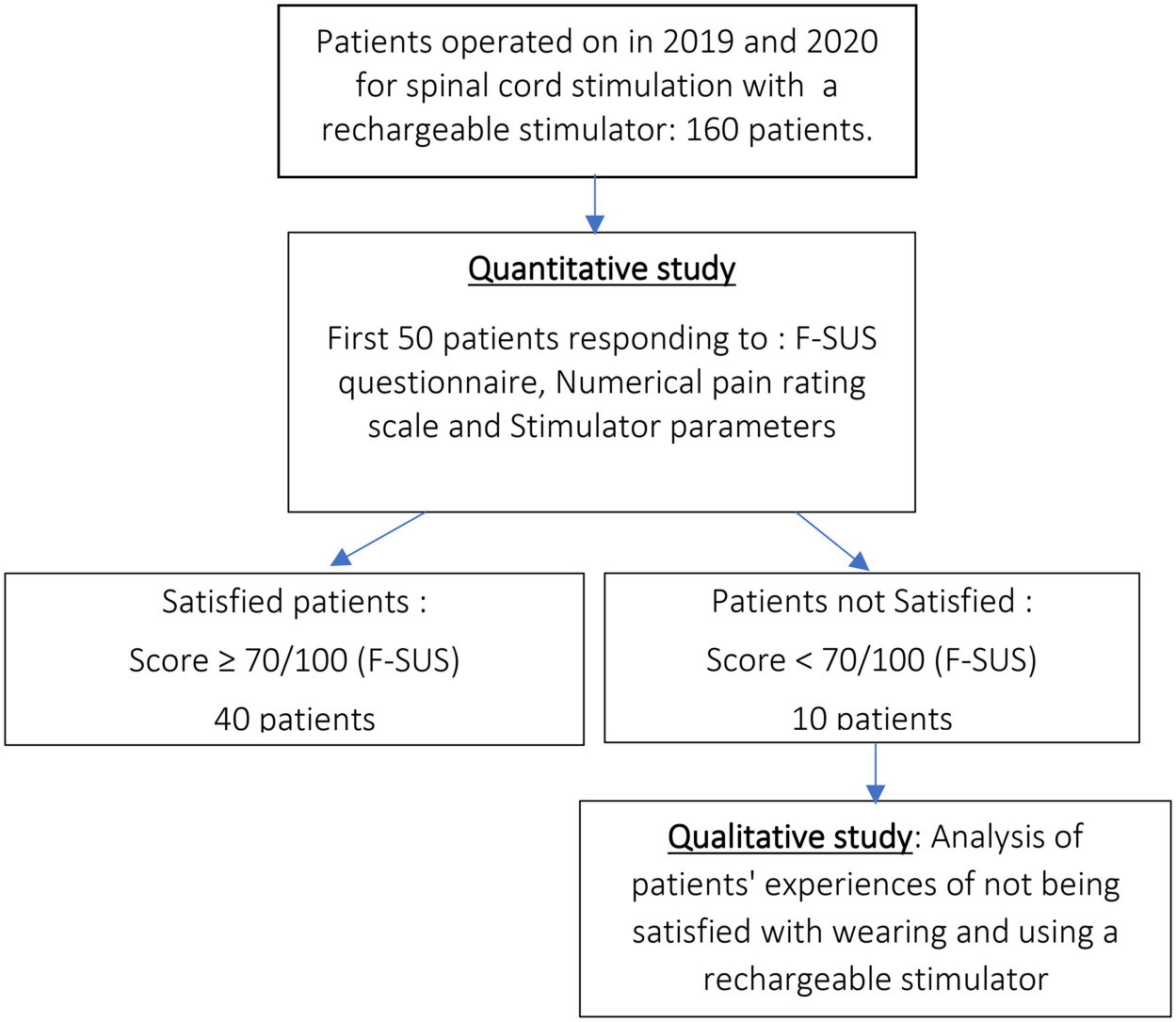
Diagram of the study.

The 50 patients included in this study corresponded to the first 50 patients who completed the questionnaires and signed the consent form (Figure 1). They were 26 women and 24 men, with a mean age of 51.6 years [+/− 9.8 (standard deviation) and extremes ranging from 32 to 75 years], operated on between January 7, 2019 and June 17, 2020 [mean follow-up of 3.6 years (+/− 2.8 and extremes ranging from 2.8 to 4.3 years) from the date of evaluation] for spinal cord stimulation using a rechargeable stimulator for the treatment of neuropathic pain refractory to medical treatment. In 42 cases, this involved dorsal (thoracolumbar) spinal cord stimulation (average level T9-T10) and in 8 cases cervical spinal cord stimulation (average level C4–C5). In 30 cases it was the first implantation (stimulation intensity greater than 5 mAmp during the one-week test period) and in 20 cases a stimulator renewal (standard stimulator life less than 3 years). In 47 cases, an Intellis® stimulator was used, in 2 cases a Prodigy® stimulator and in 1 case a Sensor® stimulator.

### Usability of the recharge procedure

The mean F-SUS score was 83.1 (+/−15.3 and extremes ranging from 42.5 to 100). Forty patients (80% of cases) had an F-SUS score ≥ 70, indicating good usability of the recharge procedure (satisfied patients' group). Of these, 26 patients had a score ≥ 90/100, reflecting excellent usability of the procedure. Ten patients (20% of cases) had an F-SUS score <70, indicating usability requiring improvement (unsatisfied patients' group). Only 1 of these patients had a score <50, indicating an “unusable” procedure (Table 1).

**Table 1:** Comparison of results between the total group of patients, the group of satisfied patients (F-SUS score ≥70) and that of unsatisfied patients (F-SUS <70) and comparison of patients with cervical spinal cord stimulation (C3-C4) and patients with dorsal (thoracolumbar) stimulation (T9-T10).

Mean(n)/SD		NRS pre	NRS Post-ST	NRS Post-LT	%Imp-ST	%Imp-LT	%Satisfac.	Duration (minutes)	Frequency (Days)	mAmp	Hz	PW	HF	HD	Tonic
Total	Mean (50 patients)	81.8	19.2	38	76	52	78.2	59.6	4.2	4.6	572.2	152.6	24	5	21
	SD	13.2	12.9	23.8	16.1	29.3	13.5	35	7.1	2.6	435.2	145.2			
*p* values (1)			<0.0001	<0.0001											
≥70	Mean (40 patients)	81.8	18.8	36.5	76.1	54	79.8	59.3	4.8	4.4	535	169.8	17	5	18
	SD	13.8	12	23.2	15.7	28.6	13.1	36.8	7.8	2.7	429.3	152.5			
*p* values (2)			<0.0001	<0.0001											
<70	Mean (10 patients)	82	21	44	75.1	45.5	72	61	1.9	5.6	721	84	7		3
	SD	11.4	16.6	26.7	18.8	32.8	13.9	28.3	1.9	1.6	449.4	86.9			
*p* values (3)			<0.0001	=0.003											
*p* values (4)					=0.464	=0.405	=0.028	=0.794	=0.397	=0.129	=0.495	=0.142			
Cerv	Mean (8 patients)	85	23.8	27.5	72.6	67.4	86.3	60	7.3	4.3	257.5	223.8	1	1	6
	SD	9.3	15.1	14.9	15.6	17.8	7.4	13.4	7	2.2	351.9	128.2			
*p* values (5)			=0.001	=0.001											
Dors	Mean (42 patients)	81.2	18.3	40	76.5	49.4	76.7	59.4	3.6	4.7	632.1	139	23	4	15
	SD	13.8	12.5	24.8	16.4	30.3	13.9	37.9	7	2.7	426.9	145.7			
*p* values (6)			<0.0001	<0.0001											
*p* values (7)					=0.528	=0.131	=0.038	=0.384	=0.004	=0.525	=0.045	=0.036			

F-SUS, French version of the System Usability Scale; NRS, numerical rating scale of pain intensity (/100) in the short term (NS Post-ST, 3 months after implantation) and the long term (NS Post-LT, several years after implantation) and the corresponding percentages of improvement (%Imp-ST, %Imp-LT). %Satisfact., the global percentage of satisfaction with the use of the rechargeable stimulator; Duration, duration of the recharge in minutes at the time of evaluation; Frequency, interval between recharges in days. Stimulation parameters, intensity (mAmp), frequency (Hz), pulse width (PW), and pattern (HF, high frequency; HD, high density; or Tonic, number of patients). SD, standard deviation; Cerv, cervical spinal cord stimulation; Dors, dorsolumbar spinal cord stimulation. Statistical analyses: p values (1): difference between preoperative and postoperative NRS scores of pain intensity for the total group of patients; p values (2): the same for the group of patients with F-SUS score ≥70; p values (3): the same for the group of patients with F-SUS score <70; p values (5): the same for the group of patients with cervical spinal cord stimulation; p values (6): the same for the group of patients with dorsolumbar spinal cord stimulation; p values (4): difference between the groups of patients with F-SUS ≥70 versus F-SUS <70 in term of %Imp, %Satisfac., duration and frequency of recharge and parameters of stimulation; p values (7): the same between the groups of patients with cervical vesus dorsolumbar spinal cord stimulation. Significant *p* values are shaded.

### Improvement in pain

The mean preoperative NRS score was 81.8 (+/− 13.2). It was 19.2 (+/− 12.9) in the short term [mean improvement 76% +/− 16.1 (*p* < 0.0001)] and 38 +/− 23.8 [mean improvement 52% +/− 29.3 (*p* < 0.0001)] in the long term. There was no statistically significant difference in short-term results between patients in the F-SUS ≥70 group (*n* = 40) and those in the F-SUS <70 group (*n* = 10) (*p* < 0.0001 in both groups). In the long term, improvement was slightly less significant in the F-SUS <70 group (*p* = 0.003) compared with the F-SUS ≥70 group (*p* < 0.0001), but there was no significant difference between the 2 groups when comparing % improvement in the long term (*p* = 0.402). This suggests that the analgesic outcome of spinal cord stimulation has no influence on the usability of the reloading system. Patients with cervical spinal cord stimulation were slightly less improved (*p* = 0.001) in the short and long term than patients with dorsal spinal cord stimulation (*p* < 0.0001) (Table 1).

### Percentage of satisfaction

Patients in the F-SUS ≥70 group were significantly more satisfied (*p* = 0.028) than patients in the F-SUS <70 group. This means that the overall impression of satisfaction with the use of a rechargeable pacemaker correlates well with the usability of the recharge system. It should be noted that patients with cervical spinal cord stimulation were significantly (*p* = 0.038) more satisfied with the recharging procedure than patients with dorsal spinal cord stimulation (Table 1).

### Recharging parameters

The average recharging time was 59.6 min (+/− 35 with extremes ranging from 15 to 210 min) (Tables 1, 2). The average interval between recharging procedures was 4.2 days (+/− 7.1 with extremes ranging from 2 times a day to 35 days). Twenty-three patients (46% of cases) recharged every day (*n* = 17) or twice a day (*n* = 6) and 27 patients (54% of cases) recharged between 2 and 35 days. We found no significant difference in the F-SUS score between patients in these 2 groups (*p* = 0.265) (Table 2).

**Table 2:** Comparison of stimulation parameters as a function of time interval between recharging procedures (≤1 day and >1 day) and duration of recharging procedure (≤1 h and >1 h).

Mean(n)/SD		F-SUS	mAmp	Hz	PW	HF	HD	Tonic
≤1 day	Mean (23 patients)	80.4	5.5	723.9	117.8	15	2	6
	SD	16.8	2.8	402.4	144.4			
>1 day	Mean (27 patients)	85.5	3.9	443	182.2	9	3	15
	SD	13.8	2.2	426.9	141.8			
*p* values (1)		0.265	0.028	0.033	0.053			
≤1 h	Mean (38 patients)	82.9	4.9	628.4	133.4	21	3	14
	SD	15.3	2.5	435.6	138.5			
>1 h	Mean (12 patients)	84	3.9	394.2	213.3	3	2	7
	SD	16.1	2.7	399.9	155.3			
*p* values (2)		0.769	0.280	0.113	0.063			

F-SUS, French version of the System Usability Scale. Stimulation parameters, intensity (mAmp), frequency (Hz), pulse width (PW), and pattern (HF, high frequency; HD, high density; or Tonic, number of patients). SD, standard deviation. Statistical analyses: p values (1): difference in stimulation parameter values between the group of patients recharging very frequently and the other patients. Higher stimulation intensities and frequencies (shaded values) are found in the group that recharges very frequently (every day or twice a day). p values (2): difference between the group of patients whose recharging procedure lasts more than an hour and the other patients. No significant differences.

Patients recharging daily used significantly higher amplitude and frequency of stimulation (*p* = 0.028 and *p* = 0.033) and used more high-frequency stimulation (*n* = 15) than patients recharging less frequently, who used more tonic stimulation (*n* = 15). This means that the parameters and type of stimulation have an influence on the frequency of recharging, but not on the usability of the recharging system (Table 1). Patients who's recharging last more than an hour use higher stimulation pulse widths (mean 213.3 µseconds) than patients with short recharging durations (mean 133.4 µseconds), with a difference close to significance (*p* = 0.063).

### Influence of stimulation site

Patients with cervical stimulation recharged significantly (*p* = 0.004) less often (on average every 7.3 days) than patients with dorsal stimulation (on average every 3.6 days). Stimulation frequencies were lower (257.5 vs. 632.1 Hz) (*p* = 0.045) and pulse widths higher (223.8 vs. 139 µseconds) (*p* = 0.036) than in the dorsal stimulation group (Table 1).

## Qualitative study

The main objective of the qualitative component of this study was to analyse in depth the different meanings associated with the complaints expressed by the 10 patients who were not satisfied with the reloading procedure (F-SUS < 70/100) (Figure 1).

### Participants

The group comprised 7 women and 3 men with an average age of 54 (+/− 11), ranging from 45 to 63. In 6 cases, this was a first-line implantation and in 3 cases a stimulator replacement. In 9 cases, the electrode was placed at the T9-T10 level and in one case at the cervical level. The average follow-up period was 3.6 years (+/− 0.6), ranging from 2.9 to 4.1 years.

### Methods of analysis

This was a “complementary use” interview survey (a survey carried out after a questionnaire survey). All patients agreed to the principle of a recorded interview and its practical arrangements and had signed the study consent form. The interviews took place individually, for the 10 patients who were not satisfied with the recharging procedure, by telephone, after a prior appointment. Each interview was recorded on a separate audio recorder. The interviews ended as planned when all 10 patients were interviewed. All interviews were conducted by the same investigator and took place between May 2023 and June 2023.

The interviews were semi-structured, with initial instruction and a thematic guide. Initial instructions: “You have a rechargeable stimulator with which you are not overly satisfied. Can you tell me about the main points of your dissatisfaction?” The thematic guide helps the interviewer to improvise relevant follow-ups on the various statements made by the interviewee; at the very moment they were addressed (20).

1. Initial explanations given to the patient.
2. Quality of training.
3. Ease of recharging.
4. Opinion on recharge time.

All interviews were transcribed verbatim and analysed to identify codes and themes (thematic analysis), following the principles outlined by Virginia Braun (27).

– CATMA (Computer Assisted Text Markup and Analysis) software was used: The coded data were imported into CATMA software, enabling the themes identified in the qualitative analysis to be structured and categorized.
– Use of IRAMUTEQ (Interface de R pour les Analyses Multidimensionnelles de Texte Et de Questionnaire) software: The coded interviews were also imported into IRAMUTEQ, providing a quantitative lexical analysis to explore lexical patterns, word associations and their frequency.
– Cross-validation between researchers: Two independent researchers (DD and G PH) validated the thematic coding selected following the various analyses.

The combined use of qualitative analysis, CATMA and IRAMUTEQ software and cross-validation creates a robust methodology that captures the complexity of the data.

Data (in French) resulting from the use of CATMA and IRAMUTEQ software are available on request (pierrehenri.garnier@chu-nantes.fr).

## Results

### Pre-implant information

Formal information: Do the patients interviewed feel that they have received information within the formal framework of their care?

How do they perceive the information provided by professionals? Do they express a need for further information?

A central finding emerges from our analysis: the discourse about formal information is ambivalent. The majority are satisfied with the information provided but express their feelings about a perceived lack of information when using the recharge system. The data below clarify and illustrate this result.

Six out of 10 patients feel they were well informed by the medical team before implantation:

P1 (patient 1) “Well informed about everything”.
P2 (patient 2) “Yes, it was very good (…), he really explained to me what was going on, what the role of the box was going to be, really, I have nothing to say (…). I completely understood the aim and purpose of what it could do”.

Four patients (stimulator replacement) said they had received little or no information, including one who said he had received explanations, but not about recharging, which for him is the most restrictive aspect of daily life.

P5 “It was clearly explained (…), the only “non-explanation” was about recharging the battery, which is extremely regular and restrictive.”
P7 “The very first one was non-rechargeable (…). So, the surprise that it was rechargeable came when I woke up (…) but we were reimplanting a stimulator because the other one was at risk of externalizing, and when I came back to the room, they explained that it was rechargeable”.

The fact of not having been educated beforehand about the recharging system and the equipment needed for recharging caused this patient a moment of panic and difficulty in accepting the therapy in the initial phase.

P8 “I didn't know there was a belt that I didn't know (…). No, I didn't know much actually”. [Interviewer] Right, and did you miss these explanations? [P8] “Yes, because when I found myself the next morning, after the aesthetic, I had to charge myself, and well, I was completely panicked, (…) I had a lot of trouble coping with the device, the recharging, all that, and I even had a breakdown, I really had a very, very bad experience”.

Informal information: Patient witnesses, acquaintances, the Internet and other sources. All the information received from patient witnesses; acquaintances and the Internet mainly concerned the spinal cord stimulation procedure and not the recharging procedure.

### Recharging

All participants reported being aware of the resources offered by the centre, such as the hotline (the company's toll-free number), the possibility of coming in to adjust, appointments with the centre's pain nurse or doctor.

P2 “I think the concept itself is good, it's well done, when you've got a problem, all you must do is phone. No, really, I have nothing to say”.
P9 “I couldn't recharge at all (…) so I went to the clinic, they tested the device and apparently my charger had a problem”.

Four patients emphasize the efficiency and confidence of the Pain Centre team and nurses.

P4 “They took care of ordering, providing me with new equipment, adjusting me, and since December it's been much better!”
P8 “They are very good, always very attentive, very responsive, very good”.

Three patients mention a difficulty in understanding the problems encountered by the hotline (problems difficult to name and locate).

P4 “I phoned Paris and (…) received a new belt (…) received a battery (…) received a new remote control”.
P6 “(…) February, breakdown again, so (…) I called again and explained what it was, so they said: “it must be the charger, or the belt” and they sent me back a belt, it was the same (…) they sent me back another one, I had to wait 15 days and it was the same (…) it didn't work at all (…) so they sent me back another charger (…) it didn't work, it wasn't the right charger they had sent me, so they sent me another one”.

### Charging system

How do patients describe ergonomic problems, and what solutions are they looking for? Ergonomic problems are described mainly in relation to recharging and the time required for recharging for 9 of the 10 patients questioned. Patients say they organize their stimulator recharging according to their daily routine and activities.

Recharging: The 10 patients said they had found ways of organizing recharging according to their daily activities.

P4 “So, I do it as soon as I'm a bit finished in my house, around 9:00–9:30 p.m., I put it on charge, the belt, the remote control, all that (…) I sit down to watch a film (…). At 10:30–11:00 p.m. last minute, when I go to bed, well it's done, I take everything off and I sleep”.
P8 “I recharge in the morning before going to work between 7:30 and 8:00 a.m. and as I still have some of the previous day's charge left, I take 20 min and the same in the evening”.

Immobility: Eight out of 10 patients say they must remain motionless to establish and maintain the connection with the charger.

P7 “(…) because as I move the sensor it beeps (…) when I'm recharging, I can't do anything”.
P9 “In fact, you mustn't move at all, otherwise it loses contact (…) and that means remaining completely static if you want the best possible charge. That's why I can't recharge during the day, because I'm busy during the day. I've tried it, it beeps all the time obviously, it loses contact (…) so I found that the evening was the best time when I was moving the least. And I had a better chance of recharging most efficiently”.

Travelling: Five patients told us they were not bothered by the recharging system when they went on vacation abroad or in France at the weekend.

P1 “I take my belt with me, well everything I need (…) it doesn't pose a problem for me”.
P7 “I don't go on vacation very much. If I go away, for example for a weekend, then I'll organize myself to do (the recharge) as close as possible to my departure and then do it again when I get back. For example, if I know I'm going away for 3 days, I know it'll last”.

The notion of constraint recurs once again in the discourse of 3 patients when they talk about organizing their vacation or leave.

P6 “Sometimes I forget to put it back to recharge, I must think about it (…) all the time (…). Even when you go away for a weekend, you can't forget to charge it, that's a constraint”.

### Material problems

Hardware problems were mentioned by 4 patients [“plate” (antenna) overheating, loose belt, broken remote control, broken charger].

P6 “(…) I find that I have a lot of breakdowns (…) I had 2 breakdowns already in November and I was without a stimulator for 15 days (…), so it was impossible to recharge, it didn't recharge at all, so they sent me (…) a charger and it worked from November to February”.
P6 “I don't find the material very solid; the belts aren't very solid (…) they're badly stitched, badly sewn (…), I had to sew it again because it wouldn't hold, the material (…) it's not solid enough”.
P8 “I did change (…) the case 5 times (…) 5 times it broke down”.

Four patients declare that they have benefited from an effective hardware solution, but that it is not always appropriate.

P3 “they changed the plate (the antenna), the belt. And I called, they changed it for me, and it charges better”.
P4 “But yes, when you phone Paris, (…) they're very theoretical up there. They send you, they send (…), they send boxes, of everything you need (…) and I even received a whole case once (…), because you see it's very theoretical in Paris, hop (…) you need (…) hop we send you (…), you'll have it the next day (…) yes but (…) well it costs all that. We don't need them to send us complete suitcases, when all we've got is a remote control that doesn't work”.

### Innovation

Four Patients would like to be able to benefit from a recharging system that does not require them to remain immobile, so that they can go about their daily business.

P7 “(…) for me, personally, what would make things easier is a recharging system (…) so that I can continue my daily routine. I mean, for example, I could say to myself: Ok, I'll put it on charge, but that won't stop me from vacuuming or cooking dinner. Ideally, no recharging”.
P9 “And ideally, I don't know if it's Wi-Fi or blue tooth, or something like that. But if there has to be connectivity, I wouldn't mind having a USB port right next to my skin so that it doesn't bother me like, you know diabetics, they've got a thing on their skin and everything, I wouldn't mind having something like that or at least, hop, I'm free to move around, I can recharge at any time of day, whether at the office or elsewhere, because at the moment, at the office, I won't be able to, so it's something that would give us greater freedom of movement”.
P10 (On the need to recharge the stimulator) “there should be a warning light (…) like your car when you run out of diesel, because I get caught very, very often (…). For me, the 2 most important things are the warning light and the size (of the charger) (…)”.

### Educational needs

What are the key terms used to describe patients' educational needs, including the reinforcement of compliance-related skills?.

For 2 patients, we note a certain difficulty in naming equipment and breakdowns.

P2 “(…) you know I've got my two, my two, what's it called? my two things (…) that say that it fills up (…)”.
P3 “the problem came from the plate (…), the belt at the level of the plate to stick on the thing, (…)”.

Reinforcement of necessary knowledge. Five patients talked about their lack of knowledge of certain elements required for recharging, given the information they had received, their difficulties in managing their recharging on a day-to-day basis, and material problems.

P3 “I didn't know there was a battery, I hadn't been told (…)”.
P4 “I didn't understand that you could put (…) the belt over your clothes, especially as I had to wash, shower and undress in my pyjamas to keep it close to my skin”.

One patient talks about her desire for individualized training (education).

P4 “(…) maybe I thought I should have taken some training. I had seen that there were little posters in the waiting room inviting people to take training courses, so I thought maybe they would have told me, (…)”.

### Satisfaction with pain relief

Four patients expressed an improvement in pain relief, despite their dissatisfaction with the recharging procedure.

P8 “Ah well, keep it up, now I'm 100% satisfied”.
P7 “It's true that in terms of pain, but it's (…) changed my life”.
P8 “Oh well, frankly, it's very good! (…) at the end of the day, it still hurts (…) it's not much, between 2 and 3 (…), because before, it never went away, never”.

Two patients mention a “loss of sense” in carrying out recharging due to the persistence of their pain.

P2 “(…) I'm in pain with the stimulator on, so I'm thinking, what's the point of recharging? That's the question I'm left with”.

### Anatomical position of the stimulator

Nine patients out of 10 implanted have a stimulator positioned at the top of the left buttock. For these 9 patients, this anatomical position does not interfere with recharging.

Nine of the 10 patients interviewed said they were not bothered by the position of the stimulator in their daily lives and did not mention any problems with their body image.

P3 “It's in the back on the left”. [Interviewer] Can you tell me, in that position, is it something that could explain why recharging is complicated? [P3] “Uh, no”.

One patient said he was bothered by the stimulator in his back because of his job as a lorry driver, for which the long sitting position becomes painful because he can feel the stimulator.

P5 “it's a nuisance, it becomes irritating in fact (…) with long periods of driving as I was telling you, and a position with pressure on the back or pressure on it (…) it becomes painful in the long run (…) over the course of a day, it's quite tiring”. The same patient specified that this did not cause any difficulty in recharging his stimulator.

## Discussion

The ease or difficulty of use of a rechargeable stimulator has already been studied in the field of spinal cord stimulation (11–13), but also in that of occipital nerve stimulation (14) or deep brain stimulation (15). In all cases, the patients were evaluated by means of questionnaires designed by the authors of the study, who were generally the surgeons who fit the stimulators. This was already an interesting approach as, until now, the evaluation of products and procedures was often based solely on the surgeon's opinion (28). The questionnaires used have not been scientifically validated either in terms of their validity (research purpose) or reliability (reproducibility of results) (29). The questionnaire used in the study of McAuley and colleagues (13) contains only 4 questions regarding usability, with 5 possible answers (from 1: poor to 5: good). In contrast, the questionnaire used Sciacca and colleagues (14) includes 36 questions with some binary answers (yes/no) and others totally free, the interpretation of which would emerge from a real qualitative analysis. Most other studies (11, 15) use a questionnaire with 24 or 25 questions and a 5-point response option in line with the Likert scale (from 1: completely disagree to 5: completely agree). In spinal cord stimulation, this disparity in the questionnaires used may explain the variability of results where the percentage of patients satisfied with the recharging procedure can range from 76 to 96%, depending on the teams and the criteria used (11–13). The use of the F-SUS questionnaire, which is dedicated to the evaluation of the usability of a procedure and validated (16), can alleviate this problem of reproducibility of results.

The F-SUS questionnaire assesses the usability of the procedure, but it is recognized that this type of questionnaire needs to be complemented by qualitative assessment methods to provide interpretable results (17). In our case, the F-SUS does not assess the different stages of the recharging procedure, nor the patients' feelings. We believe that qualitative analysis lends itself well to this evaluation. We did not find any work using a qualitative analysis of the use of rechargeable spinal cord stimulators. In contrast, several qualitative studies have been conducted on the use of non-rechargeable spinal cord stimulators (30, 31). In all cases, as in our case, the analysis was thematic and based on semi-structured interviews.

This study reveals that 80% of patients consider the recharging procedure to be satisfactory, testifying to good usability (F-SUS score ≥70/100) of this procedure. This is slightly higher than the average results in the literature, which estimate that around 25% of patients are not satisfied with the refill procedure (11–13). In our series, the duration of recharging was relatively short, averaging one hour, with extremes ranging from 15 min to 3.5 h. In the literature, the duration of a recharging session averages 2.3 h, with extremes ranging from 1 to 4 h (11, 32). A high-frequency stimulation mode with stimulation wave widths of the order of 130 µseconds would favour short recharging times (Table 2). Qualitative analysis shows that recharge duration is one of the main complaints made by dissatisfied patients. Indeed, 8 out of 10 patients say that they must remain strictly immobile, otherwise there is a loss of contact between the “plate” (the antenna) and the stimulator, and a buzzer indicates that recharging is no longer taking place. As a result, patients complain that no activity is possible during recharging. Whenever possible, patients prefer to organize short recharges (of the order of half an hour), quite often repeating them (up to 2 times a day). In this context, it's not surprising that our patients had to reload on average every 4.2 days, which is a little more frequent than what's found in the literature (average of 5.8 days) (11). Unlike the duration of recharging, the frequency of recharging sessions is not a reason for dissatisfaction among unsatisfied patients.

Equipment-related problems cannot be analysed separately in the F-SUS score, as the scale is only validated for an overall score (18). Nearly half of unsatisfied patients (4/10) reported problems with the recharging equipment. One patient had to change his charging unit 5 times. Another patient had 2 charging unit failures in the same month. These problems are also reported in the literature. Van Buyten and colleagues (12) have reported in their study that 23 of 41 patients (56.1% of patients) had a hardware problem. In addition, several unsatisfied patients found the recharging unit too bulky, which was annoying when they were out and about. These hardware problems are often resolved with the nursing staff at the centre who have taken charge of the patient and are responsible for clinical follow-up. It is therefore important to organize a follow-up that also covers breakdowns. Even patients who were dissatisfied with the recharging procedure were satisfied with the care they received at their centre in the event of a hardware problem. On the other hand, many patients were dissatisfied with the hotline provided by the company marketing the equipment.

The analgesic result of spinal cord stimulation does not seem to influence satisfaction with the recharging procedure, even if the improvement in long-term pain seems slightly less significant in the group of unsatisfied patients (*p* = 0.003) than in the group of satisfied patients (*p* < 0.0001). Some patients who were very well improved by spinal cord stimulation were able to tolerate a reloading procedure that they considered unsatisfactory (see chapter on satisfaction with pain relief).

Patient information and training was assessed in different ways. Four patients whose standard stimulator had to be replaced by a rechargeable stimulator complained that they had not been sufficiently informed about the modalities and constraints of recharging. Overall, there seems to be a lack of practical training to enable patients to familiarize themselves with the equipment and ask practical questions. This highlights the need to develop a genuine therapeutic education protocol.

As far as the equipment is concerned, patients above all want to be able to move around during the recharging procedure. Some suggested using Wi Fi or Blue tooth transmission. They find that the recharging box is too bulky and often breaks down.

It would probably have been interesting if the qualitative study had also included a series of patients who were satisfied with the recharging procedure.

This study has certain other limitations: (1) The number of 50 patients selected for the quantitative study was not based on a sample size calculation but solely on a comparison with data from the literature in the same field, based on series ranging from 25 to 41 patients (19, 21, 23). The decision to include the first 50 patients to respond could introduce a selection bias. Indeed, the patients who were most satisfied and had seen the greatest improvement from spinal cord stimulation may have responded more quickly than the others. (2) The number of 10 patients included in the qualitative study is relatively limited compared to equivalent studies, which involve an average of 13 patients (31, 33). This limitation stems from the previous point; (3) The operator's preference (SD) meant that most stimulator devices were from Medtronic®, which means that these results cannot be extrapolated to other models of rechargeable stimulators.

## Conclusion

Rechargeable stimulators improve the management of patients treated with spinal cord stimulation by reducing the number of reinterventions for stimulator replacements. In 80% of cases, patients are satisfied with the recharging procedure, as assessed by the scientifically validated F-SUS questionnaire. Qualitative evaluation indicates that the main drawback of the recharging procedure is the need to remain virtually motionless during recharging. Thus, the duration of the recharging session appeared as the main cause of dissatisfaction among patients not satisfied with the recharging procedure.

## Data Availability

The raw data supporting the conclusions of this article will be made available by the authors, without undue reservation.

## Appendix

F-SUS questionnaire (18), modified (item 1).

**Table:**

		Do not agree at all				Completely agree
1	I find this procedure unpleasant	1	2	3	4	5
2	This procedure is unnecessarily complex	1	2	3	4	5
3	This procedure is easy to use	1	2	3	4	5
4	I would need the support of a technician to be able to use this procedure	1	2	3	4	5
5	The different functionalities of this procedure are well integrated	1	2	3	4	5
6	There are too many inconsistencies in this procedure	1	2	3	4	5
7	Most people will learn to use this procedure very quickly	1	2	3	4	5
8	This procedure is very cumbersome to use	1	2	3	4	5
9	I felt very confident using this procedure	1	2	3	4	5
10	I had to learn a lot before I could use this procedure	1	2	3	4	5

For item 1, see comments in the methods chapter.

How the questionnaire is calculated: The overall score is calculated in such a way as to consider the reversed items (items 1, 2, 4, 6, 8 and 10) and to obtain a total score between 0 and 100. The calculation is done in 3 steps:

(1) First, subtract 1 point from the user's score for items 3, 5, 7 and 9 (non-inverted items).
(2) Then, for items 1, 2, 4, 6, 8 and 10 (reversed items), the value of 5 minus the score ticked by the user must be calculated for each item.
(3) The 10 new scores are added together, and the total multiplied by 2.5.
